# Structural Dynamics of HIV-1 Envelope Gp120 Outer Domain with V3 Loop

**DOI:** 10.1371/journal.pone.0037530

**Published:** 2012-05-18

**Authors:** Masaru Yokoyama, Satoshi Naganawa, Kazuhisa Yoshimura, Shuzo Matsushita, Hironori Sato

**Affiliations:** 1 Laboratory of Viral Genomics, Pathogen Genomics Center, National Institute of Infectious Diseases, 4-7-1 Gakuen, Musashi Murayama-shi, Tokyo, Japan; 2 Department of Microbiology and Cell Biology, Tokyo Metropolitan Institute of Medical Science, 2-1-6 Kamikitazawa, Setagaya-ku, Tokyo, Japan; 3 Division of Clinical Retrovirology and Infectious Diseases, Center for AIDS Research, Kumamoto University, 2-2-1 Honjo, Kumamoto, Japan; Beckman Research Institute of the City of Hope, United States of America

## Abstract

**Background:**

The net charge of the hypervariable V3 loop on the HIV-1 envelope gp120 outer domain plays a key role in modulating viral phenotype. However, the molecular mechanisms underlying the modulation remain poorly understood.

**Methodology/Principal Findings:**

By combining computational and experimental approaches, we examined how V3 net charge could influence the phenotype of the gp120 interaction surface. Molecular dynamics simulations of the identical gp120 outer domain, carrying a V3 loop with net charge of +3 or +7, showed that the V3 change alone could induce global changes in fluctuation and conformation of the loops involved in binding to CD4, coreceptor and antibodies. A neutralization study using the V3 recombinant HIV-1 infectious clones showed that the virus carrying the gp120 with +3 V3, but not with +7 V3, was resistant to neutralization by anti-CD4 binding site monoclonal antibodies. An information entropy study shows that otherwise variable surface of the gp120 outer domain, such as V3 and a region around the CD4 binding loop, are less heterogeneous in the gp120 subpopulation with +3 V3.

**Conclusions/Significance:**

These results suggest that the HIV-1 gp120 V3 loop acts as an electrostatic modulator that influences the global structure and diversity of the interaction surface of the gp120 outer domain. Our findings will provide a novel structural basis to understand how HIV-1 adjusts relative replication fitness by V3 mutations.

## Introduction

The third variable (V3) element of the human immunodeficiency virus type 1 (HIV-1) envelope gp120 protein is usually composed of 35 amino acids. The element forms a protruding loop-like structure on the gp120 outer domain [1], is rich in basic amino acids, and has aromatic amino acids for the aromatic stacking interaction with proteins. The V3 loop participates in direct binding to the entry coreceptor [2] and constitutes the most critical determinant for the coreceptor use of HIV-1 [3], [4], [5], [6]. In addition, the tip of V3 is highly immunogenic and contains neutralization epitopes for antibodies [7], [8], [9], although the epitopes can be inaccesible in the gp120 trimer on a virion of the HIV-1 primary isolates [10], [11] or HIV-1 recombinants with less positively charged V3 [12], [13]. Moreover, the V3 is reported to be the major determinant of HIV-1 sensitivity to neutralization by the soluble form of CD4 [14], [15], [16], a recombinant protein that binds to the cleft of the gp120 core [17]. Thus, the V3 loop plays a key role in modulating biological and immunological phenotypes of HIV-1. However, the molecular mechanisms underlying these modulations remain poorly understood.

It has been reported that the net charge of the V3 loop is tightly linked to the phenotype of HIV-1. The V3 loops of CCR5 tropic HIV-1s are usually less positively charged than those of CXCR4 tropic HIV-1s [18], [19], [20], [21]. An increase in the V3 net charge can convert CCR5 tropic viruses into CXCR4 tropic viruses [4], [22], [23], [24], and antibody resistant viruses into sensitive viruses [12], [13]. Thus the V3 loop may be viewed as an electrostatic modulator of the structure of the gp120 interaction surface, an assumption that is largely unexamined.

Increasing evidence has indicated that the dynamics property of molecules in solution is critical for protein function and thus for many biological processes [25], [26], [27]. Molecular dynamic (MD) simulation is a powerful method that predicts the structural dynamics of biological molecules in solution, which is often difficult to analyze by experiments alone [28], [29], [30]. Recent advances in biomolecular simulation have rapidly improved the precision and application performance of this technique [28], [29], [30]. We have previously applied this technique to investigating the structural factors that regulate biological phenotype of viruses [13], [31], [32]. In this study, by combining MD simulations with antibody neutralization experiments and diversity analysis of the viral protein sequences, we studied a structural basis for the regulation of HIV-1 phenotype by V3 loop.

## Results

### Molecular dynamics simulation study

**Figure 1:**
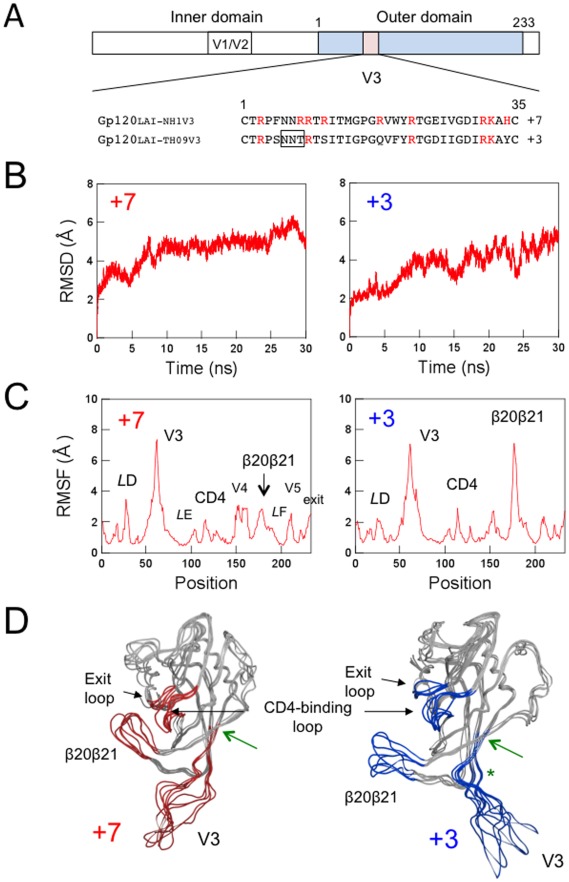
MD simulation of the identical gp120 outer domain carrying a V3 loop with net charge of +7 or +3. (A) Schematic representation of the gp120 open reading frame along with the amino acid sequences. The net charge indicates the number of positively charged amino acids (R, K, and H) minus the number of negatively charged amino acids (D and E) in the V3 loop. A light blue box indicates the outer domain used for the MD simulations. A pink box indicates the V3 loop. The numbers indicate amino acid positions at the outer domain (amino acids 1 to 233 in Figure 1A correspond to amino acids 256 to 489 in the gp120 of HIV-1_LAI_) or the V3 loop. An open black box in the V3 loop sequence indicates a potential site for the N-linked glycosylation. (B–D) Left panels: Gp120_LAI-NH1V3_; Right panels: Gp120_LAI-TH09V3_. +7 and +3 indicate the net charges of V3 loops of the recombinant proteins. (B) Time course of RMSD during MD simulations. The RMSD values indicate the structural fluctuations of the outer domain in aqueous solution. The numbers in the horizontal axes indicate the time of MD simulation. (C) Distribution of RMSF in the gp120 outer domain. The RMSF values indicate the atomic fluctuations of the main chains of individual amino acids during 10–30 ns of MD simulations. The numbers in the horizontal axes indicate amino acid positions at the outer domain. (D) Superimposition of Gp120 models at 10, 15, 20, 25, and 30 ns of MD simulation. A green asterisk indicates approximate position of a potential N-glycosylation site at the V3 stem. A green arrow indicates the site of the disulfide bond at the V3 base.

To address the potential role of the V3 net charge in modulating the structure and dynamics of the gp120 surface, we performed MD simulations of the identical gp120 outer domains carrying different V3 loops with net charges of +7 or +3 (Fig. 1A). The initial structures for the simulations were constructed by homology modeling using the crystal structure of HIV-1 gp120 containing an entire V3 loop as the template. Due to the perfect identity of the outer domain sequences of the V3 recombinant gp120s, the outer domain structures of the initial models for the MD simulations were identical before the simulations. The modeling targets in this study belong to HIV-1 subtype B and had a sequence similarity of about 87.3% to the modeling template. This similarity was high enough to construct high-accuracy models with an RMSD of ∼1.5 Å for the main chain between the predicted and actual structures in the tested cases with homology models and x-ray crystal structures [33]. These initial models were lacking in V1/V2 loops and glycans on the gp120. The recombinant models are therefore suitable for exploring the potency of the structural regulation that is intrinsic to the V3 loop.

**Figure 2:**
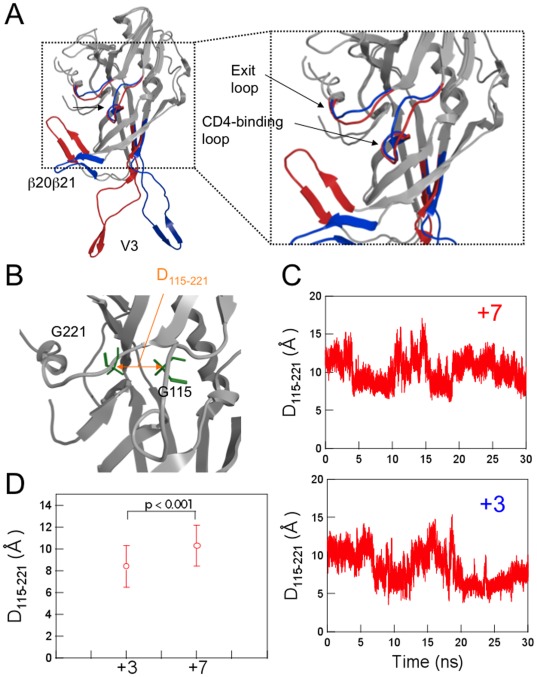
Comparison of the averaged 3-D models during MD simulation. (A) Superposition of the averaged structures obtained with the 40,000 snapshots obtained from 10–30 ns of MD simulations using ptraj module in Amber 9. Red and Blue ribbons: loops of Gp120_LAI-NH1V3_ and Gp120_LAI-TH09V3_ with V3 net charges of +7 and +3, respectively. (B–D) Configuration and structural dynamics of the CD4 binding loop. The distance between the Cα of Gly115 and the Cα of Gly221 in the CD4 binding loop was calculated to monitor configurational changes (B). The distance was monitored during the 10–30 ns of MD simulation (C) and the average distance with variance was plotted (D). +7: Gp120_LAI-NH1V3_; +3: Gp120_LAI-TH09V3_.

Using these models as the initial structures, we analyzed the structural dynamics of the gp120 outer domains in the absence of soluble CD4 by MD simulation. It was expected that the MD simulations would eliminate initial distortions in the template crystal structure, which could be generated during crystallization, and search for the most stable structures of unliganded gp120 outer domains at 1 atm at 310 K in water. The simulations showed that the same gp120 outer domains, carrying different V3 loops with net charges of +7 or +3, exhibited marked changes in conformations and fluctuations at several functional loops at 1 atm at 310 K in water (Figs. 1 and 2).

To quantitatively monitor the overall structural dynamics of the outer domain during MD simulation, the RMSDs between the initial model and models at given times of MD simulation were measured. The RMSD sharply increased soon after heating of the initial model and then gradually reached a near plateau after 10 ns of the MD simulations (Fig. 1B). The results suggested that most of the backbone heavy atoms of the outer domain reached a thermodynamic equilibrium after 10 ns of the simulation under the conditions employed. However, fluctuations of the RMSDs were still detectable even at around 30 ns of the simulations, suggesting that some regions of the outer domains continued to fluctuate.

To map the heavily fluctuating sites in the gp120 outer domain, we calculated the RMSF of the main chains of individual amino acids during the MD simulations. The RMSFs, which provide information about the atomic fluctuations during MD simulations [34], were found to be much greater in the amino acids constituting loops than those of the structured regions, such as helixes and β-sheets (Figs. 1C and 1D). These results are consistent with the general observations of proteins in solution, and indicate that the loops of the gp120 outer domain intrinsically possess structural flexibility in water. Notably, the RMSFs in some loops were markedly different between the two V3 recombinant gp120s. For example, the RMSF in the β20–β21 loop was much greater in the Gp120_LAI-TH09V3_ (Fig. 1C). Conversely, those in the D loop were greater in the Gp120_LAI-NH1V3_.

HIV-1 gp120 V3 loop often has a motif for the N-linked glycosylation that is usually preferentially conserved in R5 viruses (Fig. 1A). To address potential impacts of the glycan on the MD simulations, we performed MD simulation in the presence of a high mannose oligosaccharide in the V3 loop. We observed any significant differences in the structure and dynamics of gp120 outer domain in the presence or absence of the glycan (data not shown). This is reasonable because the glycosylation site is exposed toward an opposite direction from the gp120 core (Fig. 1D).

To clarify structural differences between the Gp120_LAI-NH1V3_ and Gp120_LAI-TH09V3_, we constructed their averaged structures using the 40,000 snapshots obtained from 10–30 ns of MD simulations using ptraj module in Amber 9. Superposition of the averaged structures showed that the relative configuration of the V3 loops and β20–β21 was markedly different between the two outer domains: the V3 tip protruded a greater distance from the β20–β21 loop in the Gp120_LAI-TH09V3_ than in the Gp120_LAI-NH1V3_ (Fig. 2A). The superposed structures also revealed differences in a region around the CD4 binding site (Fig. 2A, right panel with enlarged CD4 binding site). The relative configuration of the CD4 binding loop to the exit loop is critical for the gp120 binding to the CD4, a primary infection receptor of HIV-1 [17]. Therefore, we analyzed the distance between the CD4 binding and exit loops by measuring the distance (D_115–221_) between the Cα of Gly115 and the Cα of Gly221 as an indicator (Fig. 2B). As expected from the fluctuations of the CD4 binding loop, the D_115–221_ fluctuated during the MD simulations (Fig. 2C). However, the D_115–221_ was significantly smaller in the Gp120_LAI-TH09V3_ than in the Gp120_LAI-NH1V3_ (Fig. 2D; p<0.001, Student's *t*-test): the D_115–221_ ranged from 4–15 Å with an average of ∼8 Å for the Gp120_LAI-TH09V3_ and from 7–17 Å with an average of ∼10 Å for the Gp120_LAI-NH1V3_. These data suggest that the CD4 binding loop tended to be positioned more closely to the exit loop and thus tended to be sterically less exposed in the Gp120_LAI-TH09V3_ than the Gp120_LAI-NH1V3_.

### Neutralization study

The above structural data raised the possibility that the reduction in the V3 net charge might reduce HIV-1 neutralization sensitivity by the anti-CD4 binding site antibodies. To address this possibility, we performed a neutralization assay using the two isogenic HIV-1 recombinant viruses, HIV-1_LAI-NH1V3_ and HIV-1_LAI-TH09V3_ [35], which carry the Gp120_LAI-NH1V3_ and Gp120_LAI-TH09V3_, respectively. These viruses were pre-incubated with various human MAbs against the CD4 binding site, and the reductions in viral infectious titers were measured using a HeLa-cell-based single-round viral infectivity assay system [36].

Table 1 summarizes the results of the neutralization assay. As expected, the two viruses exhibited markedly distinct neutralization sensitivities to the three human MAbs against the CD4 binding site. HIV-1_LAI-NH1V3_ was consistently neutralized with all three MAbs against the CD4 binding site (49G2, 42F6, and 0.5δ), with ND_50_ values ranging between 0.224 and 0.934 µg/ml. In marked contrast, HIV-1_LAI-TH09V3_ was highly resistant to neutralization by these MAbs, and 10 µg/ml of antibodies failed to block the viral infections. The two viruses were equally resistant to an anti-Gp120 antibody (4C11) that recognizes the Gp120 structure after CD4 binding. The result indicates that the CD4-induced gp120 epitope of the 4C11 are not preserved in the V3 recombinant viruses used in the present study. Conversely, they were equally sensitive to another ant-Gp120 antibody (4301 [37]) whose epitope is located outside of the CD4 binding site. A human MAb 8D11 used as a negative control had no effect on the viral infectivity in this assay.

**Table 1:** Neutralization sensitivity of the isogenic V3 recombinant HIV-1 to anti-gp120 monoclonal antibodies.

Antibody ID	Ig subtype	Epitopes on Gp120	ND_50_ (µg/ml)@	
			HIV-1_LAI-NH1V3_	HIV-1_LAI-TH09V3_
49G2	human IgG1	CD4 binding site#	0.224	>10
42F9	human IgG1	CD4 binding site#	0.934	>10
0.5δ [59]	human IgG1	CD4 binding site#	0.444	>10
4C11 [59]	human IgG2	CD4 induced structure$	>20	>10
4301	mouse IgG	broadly reactive*	0.59	0.57
8D11	human IgG1	none	>20	>10

# Neutralization epitope in the Gp120 outer domain before CD4 binding.

$ Neutralization epitope induced in Gp120 after CD4 binding.

* Epitopes outside of the CD4 binding site [37].

@ The effect of each antibody on viral infectivity was tested in duplicate.

### Diversity study

Host immunity is a driving force behind the antigenic diversity of envelope proteins of the primate lentiviruses that establish persistent infection in hosts [23], [38], [39], [40], [41]. The above and previous [12], [13], [14], [15], [16] neutralization studies raised the possibility that the gp120 surface might be less heterogeneous in gp120 subpopulations that have a less positively charged V3 loop, due to greater magnitudes of resistance to the antibody neutralization. To address this possibility, we performed an information entropy study using sequences in the public database. We extracted full-length gp120 amino acid sequences of HIV-1 subtype CRF01_AE that has the same evolutionary origin and is spread throughout southeast Asia [42]), and divided them into subgroups on the basis of the net charge of V3 loop (+2, +3, +4, +5, +6, +7, and +8). The sequences were used to calculate the Shannon entropy scores, *H(i)* [1], to denote the diversity of individual amino acids within each subpopulation.

**Figure 3:**
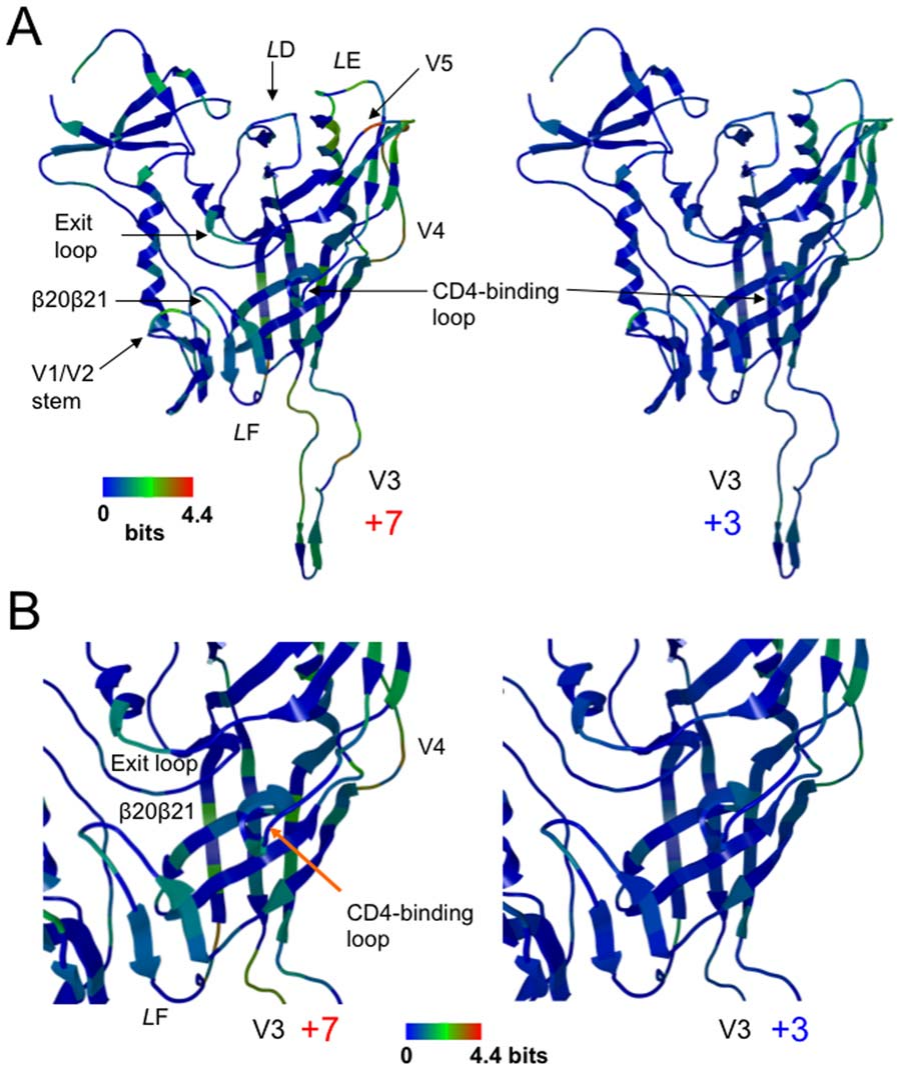
Diversity of the gp120 subpopulations carrying a V3 loop with net charge of +7 or +3. Full-length gp120 sequences of the HIV-1 CRF01_AE [42] were extracted from a public database, and divided into subgroups on the basis of the net charge of the V3 loop (+2∼+8). The divided sequences were used to calculate the Shannon entropy, *H(i)* [63], within each subpopulation, and the *H(i)* values were plotted on the 3-D structure of gp120 (PDB code: 2B4C [1]). The results for the gp120 subgroups that have V3 loops with +7 (left panel) and +3 (right panel) charges are shown as representative. The numbers of sequences used to calculate the *H(i)* were 9 and 81 for +7 and +3, respectively. (A) Distribution of *H(i)* in the gp120 monomer. (B) Distribution of *H(i)* around the CD4 binding site.

Figure 3 shows the 3-D distribution of the *H(i)* scores of individual amino acids plotted on the HIV-1 gp120 crystal structure (PDB code: 2B4C [1]), where the green to orange regions were suggested to have more variable amino acids than the blue ones. In the gp120 subpopulation that has +7 V3 loop, the *H(i)* scores often exceeded 2.0 bits at many residues, reaching close to the maximum value of 4.4, i.e., the diversity was maximal, at the V5 region (Fig. 3A, left panel). Regions with high *H(i)* scores included the functional sites, such as the V3 loop and the regions around the CD4 binding site. In marked contrast, in the gp120 subpopulation carrying the +3 V3 loop, the *H(i)* scores were almost zero, i.e., the diversity was minimal, at many amino acids, but not at those in the V4, V5, and *L*E regions (Fig. 3A, right panel). Importantly, relatively high levels of conservation were also detected with amino acids in the otherwise highly variable V3 loop. Moreover, a region adjacent to the CD4 binding loop was also less heterogeneous compared with those of the gp120 subpopulation carrying +7 V3 loop (Figs. 3B). In the gp120 subpopulations carrying the +2, +3, 4, and +5 V3 loops, the *H(i)* scores were indistinguishable from each other: they were less heterogeneous than the subpopulations carrying the +6, +7, and +8 V3 loops. Similar results were obtained with HIV-1 subtype C that represents the most predominated HIV-1 in the world (data not shown).

## Discussion

The ability of HIV-1 to rapidly change its phenotype greatly complicates our efforts to eradicate this virus. Elucidation of structural principles for the phenotypic change may provide a clue to control HIV-1. In this study, by combining MD simulations with antibody neutralization experiments and diversity analysis of the viral protein sequences, we studied a structural basis for the phenotypic change of HIV-1 by V3 mutations. To address this issue, we used a V3 recombinant system; we performed a computer-assisted structural study and an infection-based neutralization assay using gp120 proteins whose amino acid sequences are identical except for V3 loop. In combination with an informatics study, we obtained evidence that the HIV-1 V3 loop acts as an electrostatic modulator that influences the global structure and diversity of the interaction surface of the gp120 outer domain.

Using MD simulation, we first examined whether the V3 net charge could affect the structural dynamics of the HIV-1 gp120 outer domain surface. Initial structures of the outer domain of the two gp120s, Gp120_LAI-NH1V3_ and Gp120_LAI-TH09V3_, were identical before MD simulations, because the domains were both derived from HIV-1_LAI_ strain. Remarkably, however, the two molecules with distinct V3 loop exhibited markedly distinct structural dynamics following MD simulations (Figs. 1 and 2). These data strongly suggest that the V3 net charge can act as an intrinsic modulator that influences the structural dynamics of the interaction surface of the gp120 outer domain. Such a global effect on structure by a local electrostatic change has been reported with bacteriorhodopsin [43]. In general, the long-range effects of non-electrostatic contributions are negligible, whereas those of the electrostatic contributions are not [34]. Therefore, it is reasonable that the changes in overall charge of the V3 loop element caused the global effects on the gp120 structure via alteration of the electrostatic potentials of the gp120 surface.

We next studied biological impact of the structural changes predicted by MD simulations. The MD simulations suggested that the CD4 binding loop was less exposed in the Gp120_LAI-NH1V3_ than the Gp120_LAI-NH1V3_ (Fig. 2). The finding predicted that reduction in V3 net charge could cause reduction in neutralization sensitivity to the anti-CD4 binding site antibodies. This possibility was assessed by neutralization assay. We used infectious HIV-1_LAI_ clones having the Gp120_LAI-NH1V3_ or the Gp120_LAI-TH09V3_ to assess their neutralization sensitivities to the anti-CD4 binding site MAbs. Notably, we indeed observed marked reduction in the neutralization sensitivity in HIV-1_LAI_ having Gp120_LAI-TH09V3_ (Table 1). The results are consistent with the structural changes predicted by MD simulations, as well as previous findings on neutralization sensitivity of HIV-1s to soluble CD4 [14], [15], [16].

We further studied evolutionary impact predicted by MD simulations and the neutralization studies. These studies predicted that reduction in V3 net charge could cause reduction in sequence diversity around the CD4 binding site due to reduced sensitivity to positive selection pressures of antibodies. Notably, we indeed observed marked reduction in the gp120 diversity: our Shannon entropy data show that otherwise variable surfaces of gp120, such as V3 and a region around the CD4 binding loop, are less heterogeneous in the gp120 subgroups carrying a V3 loop with a +3 charge (Fig. 3).

Previous cryo-electron microscopy studies have indicated that gp120 forms a trimer on an HIV-1 virion, where the CD4 binding sites are exposed on the outside surface in the solution [44], [45], [46]. Therefore, it is reasonable that gp120 with +3 V3 with less exposed CD4 binding loop is less sensitive to neutralization by anti-CD4 binding site antibodies (Table 1) and less heterogeneous around the CD4 binding site (Fig. 3). Collectively, our results obtained with all three approaches agree with each other and suggest that V3 net charge is an intrinsic factor that influences structural property, antibody sensitivity, and sequence diversity of CD4 binding site.

The HIV-1 gp120 outer domain has several functional or immunogenic loops involved in binding to CD4, coreceptor and antibodies. Our MD simulations predicted that V3 net charge influences fluctuation and conformation of these loops (Figs. 1 and 2). The V3-based structural modulation of the gp120 surface loops may be an effective mechanism to alter effectively the phenotype and relative fitness of HIV-1. For example, a change in the V3 net charge by mutations may induce changes in V3 conformation (Figs. 1D and 2A) [13], which in turn may influence intra- or inter-molecular interactions among gp120 monomers and thus global structure of gp120 trimer on a virion. Generation of a swarm of structural variants by V3 mutations could help generating the best-fit variants under changing environments during persistent infection of HIV-1 *in vivo*. Further study is necessary to address above issue.

Thus far fine structures of neither the intact gp120 monomer nor trimer are available. However, recent crystal structure study disclosed a structure of V1/V2 domain [47], which had been the major gp120 region lacking structural information. The V1/V2 domain is located on the outer surface of gp120, as is V3, and can participate in phenotypic changes of HIV-1 [48], [49]. In this regard, Kwon et al [50] have found an intriguing role of gp120 variable loops; gp120 core has an intrinsic preference to form the CD4-bound conformation, whereas the variable loops, such as V1/V2 and V3 loops, play key roles in preventing conformational transitions into the CD4-bound state that is sensitive to neutralization. Thus it is conceivable that configurational changes of V3 loop by V3 mutations play roles in modulating structural dynamics of the unliganded gp120 core and neutralization sensitivity of HIV-1. Availability of the V1/V2 loop structure will promote structural study of the whole gp120 monomer containing V3 loop, V1/V2 domain, and glycans. Our findings will provide a structural basis to elucidate intra-molecular interactions of these elements, which in turn will allow the study of structure, function, and evolution of gp120 trimer. Incorporation of MD simulation into these studies will help understanding structural dynamics with which HIV-1 adjusts its relative replication fitness in nature.

## Materials and Methods

### Characteristics of the gp120 proteins and HIV-1s used

We used two isogenic recombinant gp120 proteins, termed Gp120_LAI-NH1V3_ and Gp120_LAI-TH09V3_ [35], for the present structural and neutralization studies. They differ only in their V3 loops. The Gp120_LAI-NH1V3_ and Gp120_LAI-TH09V3_ have the 35-amino-acid-length V3 loops from HIV-1-infected individuals in the gp120 backbone of the HIV-1_LAI_ strain [35]. The net charges of the V3 loops are +7 and +3 for the Gp120_LAI-NH1V3_ and Gp120_LAI-TH09V3_, respectively (the V3 net charge represents the number of positively charged amino acids (R, K, and H) minus the number of negatively charged amino acids (D and E) in the V3 loop). The HIV-1_LAI_ carrying the Gp120_LAI-NH1V3_ (HIV-1_LAI-NH1V3_) is the CXCR4 tropic virus, whereas that carrying the Gp120_LAI-TH09V3_ (HIV-1_LAI-TH09V3_) is the CCR5 monotropic virus [35]. The HIV-1_LAI-NH1V3_ is sensitive to neutralization by antibodies with the ability to bind to the peptides containing the autologous V3 tip sequences, whereas HIV-1_LAI-TH09V3_ is highly resistant to antibodies targeting the autologous V3 tip sequences [13].

### MD simulation

As the initial structures for the MD simulation, we first constructed three-dimensional (3-D) models of the outer domains of the Gp120_LAI-NH1V3_ and Gp120_LAI-TH09V3_ by the comparative (homology) modeling method (reviewed in [33], [51], [52]), as described previously [13]. We used the crystal structure of HIV-1 gp120 containing an entire V3 region at a resolution of 3.30 Å (PDB code: 2B4C [1]) as the modeling template. The gp120 core is in complex with the CD4 receptor and the CD4 induced structure (CD4i) antibody X5 [1]: it represents the structure after the CD4 binding. We deleted the structures of the CD4 receptor and the X5 antibody from the 2B4C complex structure to construct the free gp120 outer domain models of HIV-1_LAI_ V3 recombinant viruses by homology modeling. Then the models were subjected to the MD simulations to analyze structure and dynamics of the gp120 outer domain in the absence of the CD4 receptor and the X5 antibody interactions. The homology modeling was performed using tools available in the Molecular Operating Environment (MOE) program (MOE 2008.10; Chemical Computing Group Inc., Montreal, Quebec, Canada). The 186 amino-terminal and 27 carboxyl-terminal residues were deleted to construct the gp120 outer domain structure. We optimized the 3-D structure thermodynamically via energy minimization using an MOE and an AMBER99 force field [53]. We further refined the physically unacceptable local structure of the models based on a Ramachandran plot evaluation using MOE. MD simulations were performed as described previously [13] using the Sander module in the Amber 9 program package [54] and the AMBER99SB force field [55] with the TIP3P water model [56]. Bond lengths involving hydrogen were constrained with the SHAKE algorithm [57], and the time step for all MD simulations was set to 2 fs. A nonbonded cutoff of 12 Å was used. After heating calculations for 20 ps to 310 K using the NVT ensemble, the simulations were executed using the NPT ensemble at 1 atm at 310 K for 30 ns. Superimpositions of the Gp120_LAI-NH1V3_ and Gp120_LAI-TH09V3_ structures were done by coordinating atoms of amino acids along the β-sheet at the gp120 outer domain. We performed two independent MD simulations with distinct MD codes and obtained similar results. Therefore, we present here the data set from one of the MD simulations as a representation.

### Calculation of the root mean square deviation (RMSD) and root mean square fluctuation (RMSF)

The RMSD values between the heavy atoms of the two superposed proteins were used to measure the overall structural differences between the two proteins [34]. We also calculated the RMSF to provide information about the atomic fluctuations during MD simulations [34]. In this study, we calculated the RMSF of the main chains of individual amino acids using the 40,000 snapshots obtained from MD simulations of 10–30 ns. The average structures during the last 20 ns of MD simulations were used as reference structures for the calculation of the RMSF. Both the RMSD and RMSF were calculated using the ptraj module in Amber 9 [34].

### Monoclonal antibodies (MAbs)

The 49G2, 42F9, 0.5δ and 4C11 antibodies used for the neutralization assay were the human MAbs established from an HIV-1-infected patient with long-term non-progressive illness. Human blood samples were collected after signed informed consent in accordance with study protocol and informed consent reviewed and approved by Ethics committee for clinical research & advanced medical technology at the Faculty of Life Science Kumamoto University. B cells from the patient's peripheral blood mononuclear cells were transformed by EBV, followed by cloning as described previously [58]. The culture supernatant from an individual clone was screened for the reactivity to gp120_SF2_ by an enzyme-linked immunosorbent assay (ELISA). The specificity of antibodies was determined by gp120 capture ELISA and FACS analysis as described previously [59]. Briefly, reactivity of the mAbs against monomeric gp120 of HIV-1_SF2_ was measured with a gp120 capture assay in the absence or presence of soluble CD4 (0.5 µg/ml). Decrease in the binding activity was observed for the mAbs 0.5δ, 49G2, and 42F9 in the presence of soluble CD4, whereas enhancement in the reactivity was detected for the mAb 4C11. Reactivity of the mAbs against envelope protein on the cell surface was measured with a FACS analysis of PM1 cells chronically infected with JR-FL in the absence or presence of soluble CD4 (0.5 µg/ml). No significant difference was observed for the binding profiles of 0.5δ, 49G2, and 42F9 in the presence of soluble CD4, whereas marked enhancement of binding was observed for the 4C11 in the presence of soluble CD4. Based on these binding data, we classified 49G2, 42F9, and 0.5δ as CD4 binding site Mabs, and 4C11 as a CD4-induced epitope. All MAbs used in this study were purified by affinity chromatography on Protein A Sepharose. A human MAb 8D11 was used as a negative control for the neutralization assay. Mouse MAb 4301 was purchased from Advanced BioScience Laboratories, Inc. (Kensington, MD). The 4301 was raised with a mixture of purified gp120 of HIV-1_IIIB_ and HIV-1_MN_ and broadly reactive with the gp120 of different HIV-1 isolates [37].

### Neutralization assay

We used the two above-described V3 recombinant HIV-1s, HIV-1_LAI-NH1V3_ and HIV-1_LAI-TH09V3_ [35], for the neutralization study. The HIV-1 cell-free viruses were prepared by transfection of the plasmid DNAs into HeLa cells as described previously [24], [35], [60]. The neutralization activities of antibodies were measured in a single-round viral infectivity assay using CD4^+^CXCR4^+^CCR5^+^ HeLa cells [36] as described previously [13]. Briefly, equal infectious titers of viruses (300 blue-cell-forming units) were incubated with serially diluted MAb preparations (0.03–10 µg/ml) for 1 hour at 37°C. The cells were infected with the virus-antibody mixture for 48 hours at 37°C, fixed, and stained with 5-bromo-4-chloro-3-indolyl-β-D-galactopyranoside. Each antibody dilution was tested in duplicate, and the means of the positive blue cell numbers were used to calculate the 50% inhibition dose of viral infectivity (ND_50_).

### Analysis of amino acid diversity

Amino acid diversity at individual sites of the HIV-1 gp120 sequences was analyzed with Shannon entropy scores as described previously [13], [61], [62]. Full-length gp120 amino acid sequences of the HIV-1 subtypes CRF01_AE and C were obtained from the HIV Sequence Database (http://www.hiv.lanl.gov/content/sequence/HIV/mainpage.html). The sequences were divided into subgroups based on the net charge of V3 loop (+2∼+8) using a software system, InforSense 5.0.1 (InforSense Ltd. http://www.inforsense.com/); arginine (R), lysine (K), and histidine (H) were counted as +1, aspartic acid (D) and glutamic acid (E) as -1, and other amino acids as 0. The numbers of sequences used for the analysis of CRF01_AE were 11, 81, 57, 28, 18, 9, and 4 for +2, +3, +4, +5, +6, +7, and +8, respectively. The amino acid diversity within each V3 subpopulation of the same HIV-1 subtype was calculated using Shannon's formula [63]:
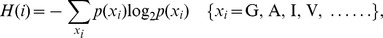
where *H(i)*, *p(x_i_)*, and *i* indicate the amino acid entropy score of a given position, the probability of occurrence of a given amino acid at the position, and the number of the position, respectively. An *H(i)* score of zero indicates absolute conservation, whereas 4.4 bits indicates complete randomness. The *H(i)* scores were displayed on the 3-D structure of an HIV-1 gp120 (PDB code: 2B4C [1]).
